# Factors influencing selection of anesthesiology as a career specialty by residents in Postgraduate Medical Institute Quetta

**DOI:** 10.12669/pjms.42.1.12256

**Published:** 2026-01

**Authors:** Muhammad Iqbal Khan, Ambareen Khan, Ambreen Usmani

**Affiliations:** 1 Muhammad Iqbal Khan Associate Professor of Surgery, Postgraduate Medical Institute Quetta, Pakistan; 2 Ambareen Khan Assistant Professor of Obstetrics and Gynecology, Aga Khan University Hospital Karachi, Pakistan; 3 Ambreen Usmani Principal and Professor of Anatomy, Sindh Medical College, Jinnah Sindh Medical University, Karachi, Pakistan

**Keywords:** Anesthesiology, Career, Influencing factors, Residents, Specialization

## Abstract

**Objectives:**

To determine the factors influencing the selection of anesthesiology as a future career by the residents at Postgraduate Medical Institute Quetta (PGMIQ).

**Methodology:**

After obtaining ethical approval from Post Graduate Medical Institute (PGMI), Quetta this sequential mixed-method study was conducted for a period of six months from 1^st^ of January, 2024 to 30^th^ June, 2024, to investigate the factors influencing the selection of anesthesiology by Postgraduate residents. In the first phase 36 postgraduates were administered a questionnaire to quantify the influencing factors. Chi-square tests (p<0.05) assessed gender associations. This was followed by a second phase in which in-depth interviews were conducted to get the qualitative insight of the first quantitative phase.

**Results:**

Out of 36 residents of PGMIQ, 19 (53%) showed personal interest, 11 (31%) showed mentor influence as the primary motivating factor. As far as practice was concerned, 33(91%) preferred practice in academic facility and 26 (72%) were motivated to integrate research in their future practice. It was also noted that 33 (91.7%) were planning for specialization in subspecialities of anesthesia with significant gender association. The plan for future practice varied, 18(50%) were planning to pursue in same institution, 4(11%) wanted to move to different institutions but within the province, whereas 5(13.9%) intended to move to other provinces and 9(25%) wanted to leave for abroad. During Qualitative phase, four key themes were revealed. First theme was career motivation which was driven by global deficiency of anesthetists and lifesaving aspects of anesthesia during difficult procedures. Second theme emerged as the professional deterrent “inequity & dependency” which was supported by their response as poor remuneration, dependency on surgeon and stigmatized labels like “نشے والا ڈاکٹر”. Thirdly, Systemic improvements were suggested, such as student mentorship and government support for subspecialties and equipment. Finally, Professional Standards emphasized the urgent need to ban unqualified practitioners to ensure patient safety and elevate the specialty.

**Conclusion:**

Analysis revealed that the primary driving factor in selecting anesthesiology as a specialty was the residents’ intrinsic fascination with the field, coupled with their desire to pursue a career in research and academia.

## INTRODUCTION

Balochistan is the largest province of Pakistan with a scattered population. Its healthcare infrastructure includes 29 District Headquarter hospitals and five major teaching hospitals in the provincial capital. However, these facilities are serviced by only 25 qualified anesthetists.^1^ Anesthesiologists provide critical care in ICUs, delivering Basic Life Support (BLS) and Advanced Cardiac Life Support (ACLS), in addition to their traditional duties in operation theaters.^2^ This critical specialty has faced a significant worldwide shortage for decades.^3^

The selection of a medical specialty by graduates is a primary determinant of the future healthcare workforce. Proper planning and a balanced distribution of graduates across all specialties are vital; failure in this planning results in the waste of financial and human resources, leading to an imbalanced and faulty healthcare system.^4^ In Baluchistan, the healthcare system faces a severe deficiency of anesthetists, a condition that is worse than many other regions. Despite the critical nature of this shortage, very little work has been done in Pakistan, and particularly in Baluchistan, to investigate the factors influencing the choice of specialty. As far as anesthesiology is concerned, no prior study has been conducted in the province

The selection of a specialty is influenced by a combination of intrinsic, extrinsic, and situational factors. These can include personality traits and personal experiences according to Kalimesetty et al.^5^, working hours, controllable lifestyle as mentioned by Nakayasu et al.^6^ and Hohf- Hohf-Nova M et al.^7^, income and years of training as reported by Al-Ansari SS et al.^4^ Orbach-Zinger et al.^8^ and Tyagi et al.^9^ argued that postgraduates are more influenced towards economic benefits, and on the contrary, Khan J et al 10 argues that it is the personal interests that are strong influencers in choosing anesthesiology. Bosco L et al. on another hand reported that female graduates are more inclined to opt anesthesiology as career specialty.^11^

This study explored the factors motivating trainees to choose anesthesia as a specialty at the Postgraduate Medical Institute Quetta, Balochistan. The knowledge of these motivational factors will help the policymakers, medical educationists and institutional leaders to develop strategies to attract more postgraduates for anesthesia department and to upgrade training programs for resource limited areas like Balochistan. Ultimately, supporting the development of the regional workforce and enhancing the understanding of specialty selection in Pakistan.

## METHODOLOGY

This two-phased sequential mixed-methods study was conducted at Post Graduate Medical Institute (PGMI), Quetta over a period of six months from 1^st^ January 2024 to 30^th^ June 2024.

### Ethical Approval:

It was obtained from the Institutional Ethical Review Committee of PGMIQ (Ref. No. PGMI/ERC/1/2023; dated December 6, 2023).

A sample of 36 residents was included in this mixed-method study based on inclusion criteria. Individuals who have passed either the FCPS-I examination in anesthesiology or the admission test for the PGMIQ diploma/MS program in anesthesiology, and have commenced their training in the anesthesiology department, will be referred to as residents. Specialty refers to a certain discipline of medical science where patients receive specialized treatment from doctors who have dedicated their careers to that field. In the first phase, the residents enrolled in PGMIQ for anesthesiology were given a questionnaire to find out the quantitative frequencies of influencing factors and in the second phase, qualitative interviews of these residents were conducted for a better explanation of the quantitative results. Informed consent was taken prior to each phase.

The first phase was intended to measure quantitatively the factors affecting the choice of anesthesia and in the second phase, in-depth analysis of these factors was done qualitatively. For both quantitative phase (QUAN) and qualitative (QUAL) phase purposive sampling was followed and the postgraduate residents of anesthesia available in PGMIQ in first-year residency as well as newly selected residents were included in the study.^12^ Data was collected on a pre-formed questionnaire for the QUAN phase. This quantitative questionnaire was designed to evaluate factors influencing postgraduate trainees’ selection of anesthesiology as a specialty. It collected demographic data and assessed career intentions, motivators (including personal interest, mentorship, and lifestyle considerations), and professional aspirations such as sub-specialization and research integration. These items directly correlate respondents’ influences and future practice preferences with their interest in pursuing a career in anesthesiology. For the questionnaires two articles were studied.^10^,^13^ The research work of Izgi M et al. was followed to develop the questionnaire with few modifications after permission from the principal author.^13^ Once it was developed it was checked by two anesthesiologists and one medical educationist for validation. After receiving their comments, the questionnaire was subjected to Cronbach’s Alpha to check its reliability. For this purpose, 20 residents were requested to fill out the said questionnaire. Cronbach’s Alpha was calculated using SPSS version 20, yielding a value of 0.848, which is considered acceptable. After gathering the quantitative data, it was entered into SPSS for analysis. A Chi-Square test was then conducted to assess the association between gender and residents’ opinions on various aspects, with significance evaluated at the 0.05 level.

For the second phase of qualitative (QUAL) analysis data was collected after explaining the study’s aim and obtaining informed consent from participants who volunteered to be interviewed. Following consent, the time, venue, and date of each interview were scheduled in advance with each participant. During the interviews, three key questions were asked:

- What motivated you to join the Department of Anesthesia?
- Why do you think people often overlook Anesthesia as a specialty, and conversely, what drives others to choose it as their career?
- How can the Department of Anesthesia be enhanced to better meet the needs of postgraduates, as well as the needs of our province and country?

Qualitative interviews were conducted by an experienced researcher with strong expertise in qualitative methods. Data collection continued until thematic saturation was achieved. Each interview, which lasted approximately 60 to 65 minutes, was audio-recorded and subsequently transcribed verbatim. The transcripts were then translated into English for analysis.

A thematic analysis was employed to identify key patterns and themes. This involved manually coding the transcripts through an iterative process, where statements were systematically grouped into emerging themes. The coding procedure and themes were checked by two medical educationists who were proficient in qualitative research. The credibility was further enhanced by member checking and sharing summarized findings with participants for verification and feedback. To ensure confidentiality and security, the data was stored on password-protected computers, accessible only to the primary researcher.

## RESULTS

A total of 36 residents participated in the QUAN phase. The program distribution was: FCPS, 4 (11.11%); MS, 8 (22.22%); MCPS, 6 (16.67%); and Diploma, 18 (50%). The cohort included 20 (55.6%) males and 16 (44.4%) females, with 26 (72.2%) being freshly inducted and 10 (27.8%) in their first year. All 36 participants (100%) intended to pursue a career in Anesthesia.

The gender-wise association between the participants’ interest in specialization and their preference for research integration is shown in Table-II A significant association was found for both males (chi sq value =10.371, p=<0.000) and females (chi sq value =23.429, p=<0.000). Among males (n=20), 19 preferred to specialize, and 12 of those 19 also preferred to integrate research. Among females (n=16), 14 were willing to specialize, and 13 of those 14 preferred research integrations, while two female postgraduates were either unwilling or unsure about research.

**Table-I:** Participants’ Motivation and Future Planning.

Information source	Freq	%
PGMIQ website	28	77.8%
Website of society of Anesthesiology	7	19.4%
Staff	1	2.8%
***Participants’ Motivation***		
Personal interests	19	53%
Mentor	11	30.6%
Academic career interest	2	5.6%
Family/ personal issues	2	5.6%
Lifestyle	2	5.6%
***Practice Preferences***		
Academic Facilities	33	91.7%
Community Hospitals	2	5.6%
Private Clinics	1	2.8%
***Future Practice***		
Integrating Research	26	72.3%
Did not want to integrate research	6	16.6%
Uncertain about Research	4	11.1%
***Future Specialization***		
Subspecialties of Anesthesia	33	91.7%
Don’t want to continue	1	2.7%
Uncertain about Decision	2	5.6%

**Table-II:** Gender-wise cross-tabulation Specialization in anesthesia & Prefer Research in Practice & Site of Practice.

Gender of Participant			Would Prefer Research in your Practice			Total	Chi sq	df	p-value	
			Yes	No	Unsure					
Male	Specialization in a subspeciality of Anesthesia	Yes	12	5	2	19	10.371	2	0.000	
		No	0	0	0	0				
		Unsure	1	0	0	1				
	Total		13	5	2	20		
Female	Specialization in subspeciality of Anesthesia	Yes	13	0	1	14	23.429	4	<0.000	
		No	0	1	0	1				
		Unsure	0	0	1	1				
	Total		13	1	2	16		
Total			26	6	4	36		
*Gender of Participant*			*Site of Future Practice*							
			*Same institute*	*In the province different institution*	*Out of the province*	*Abroad*	*Total*	*Chi sq*	*df*	*p-value*
Male	Specialization in a subspeciality of Anesthesia	Yes	8	1	3	7	19	1.287	3	0.732
		Unsure	1	0	0	0	1			
	Total		9	1	3	7	20			
Female	Specialization in subspeciality of Anesthesia	Yes	9	3	0	2	14	16.000	6	0.014
		No	0	0	1	0	1			
		Unsure	0	0	1	0	1			
	Total		9	3	2	2	16		
Total			18	4	5	9	36		

Only 10 residents were interviewed in the QUAL phase, the data of which is represented in Table-III. Four themes emerged on thematic analysis, theme one driving career growth & Saving lives. The residents were inspired to join the anesthesiology department due to the global lack of anesthetists and the readily available career opportunities. Furthermore, several individuals were motivated to work in ICU. One participant quoted, ***“I love it when i am called to do difficult intubations and when I save lives***”.

**Table-III:** Results of QUAL phase.

Themes & subthemes	Codes	Number of Responses	Verbatim Comments
**Theme: Driving career growth & Saving lives**. Easy job availability. Lifesaving procedures.	Worldwide need Lifesaving techniques	6 7	“Globally lack of anesthetists and i can get the job easily” “I love it when i am called to do difficult intubations and when I save lives.”
**Theme: Inequity & Dependency.** Insufficient remuneration. Derogatory language	Unfair compensation Professional disrespect	6 7	“I feel dependent on surgeons for my payments.” “I felt bad whenever patients and their relatives call me **“نشے والا ڈاکٹر”**
**Theme: Pioneering & uplifting anesthesia practice.** Counseling Innovation & excellence	Early guidance Advancing the Field	8 7	“I think the students in M.B.B.S should be mentored to join anesthesia” “Introduce subspecialties in anesthesia and enhance the availability of advanced equipment for the anesthesia and pain management.”
**Theme: Professional standards and regulation.**	Standardization	9	“Governments should not allow unqualified individuals to practice as anesthetists”

The second theme emerged from residents’ belief that most doctors and residents were reluctant to join the anesthesia department due to the perceived poor remuneration and reliance on surgeons for private practice. ***“I feel dependent on surgeons for my payments”*.**

Furthermore, another deterrent to pursuing a career in anesthesiology is the prevalence of derogatory language used to refer to anesthetists within the medical community like **(نشے والا ڈاکٹر)** *which could mean the “doctor who gives drugs “or “a drugged doctor” in direct English translation*.

The third theme Pioneering and uplifting anesthesia practice emerged with residents’ suggestions for implementing counseling/ mentoring for M.B.B.S students as a means of improving the anesthesia department. In addition, the participants emphasized that the government should implement significant measures to introduce subspecialties in anesthesia and enhance the availability of advanced equipment for the anesthesia department and pain management.

The final theme, professional and standard practice, emerged with residents’ belief that to enhance the quality of residency in Anesthesia, the government should prohibit unqualified individuals from practicing in both public and private hospitals. “***Governments should not allow unqualified individuals to practice as anesthetists*.**”.

## DISCUSSION

Our study indicates that intrinsic motivation is the primary driver for opting for anesthesiology at PGMIQ, a trend that aligns with global research findings. The quantitative data revealed that personal interest was the foremost reason for specialty choice, a finding that is well explained in qualitative theme of “saving lives” and the rewards for performing difficult ICU procedures like intubations. The predominance of personal interest is also reported by Khan J et al that suggests that despite contextual differences, the main attraction towards the field of anesthesia is its hands-on, critical care nature.^10^ On flip side extrinsic factors like mentor influence, lifestyle and academic career played secondary role as was also observed by Roberts LJ et al.^14^

Aside from intrinsic and extrinsic factors, availability of Jobs and academic pursuit emerges as a significant motivation. The qualitative theme of “global lack of anesthetists” shows that postgraduate residents at PGMIQ are aware of high chances of employability in anesthesia. This can be a powerful incentive for a province like Balochistan with critically deficient workforce. Similar findings were reported by Bosco L et al. and Bissing MA et al. 11,15 Majority, 91.7% of the residents were inclined to pursue sub-specialization followed by 72% who were enthusiastic about integration of research into their future practice. These findings exceed the 35.2% findings reported by Izgi et al.^13^ in Turkey, however our findings are consistent with findings of Bosco L et al.^11^ This finding may be due to abundant opportunities of career advancement in unexplored subspecialities of anesthesia in Balochistan, a need which is also highlighted by the residents who called to “introduce subspecialties” in qualitative phase. Yet the alarming finding was that 25% of the residents wanted to leave the country, which highlights that the local infrastructure should evolve to reduce the risk of brain drain.

A near equal distribution among both genders with significant association between gender and future preferences for sub-specialization was seen in this study. These findings align with the studies by Lorello GR and Knoll MR, which shared gender parity in the field of anesthesia.^16^,^17^ This could be due to rising number of female students pursuing M.B.B.S and specifically in Balochistan due to socio-cultural context, where anesthetist due to inherited specialty structure works in close environment of operating theater having limited interaction with public and patient’s attendants making this field attractive for female doctors.

Aside from above this study also revealed several obstacles which align with global challenges. While Kandikar L et al.^18^ reported lack of recognition, the qualitative data of this study showed more severe manifestations that include “insufficient remuneration”, sense of “financial dependency on surgeons” and ingrained professional disrespect **“نشے والا ڈاکٹر”** which creates a unique socio-economic barrier that deter potential talent.

The decision to chase anesthesiology as a career specialty at PGMIQ is a complex interplay of internal motivation like professional and academic ambition, against severe deterrents like financial dependency and discredit. To effectively attract and retain talent, strategic interventions must be followed: amplifying the positive motivators through mentorship, subspecialty development and advancement in training for BLS/ ACLS etc.^19^ while simultaneously addressing the negative factors by improving financial status, ensuring professional autonomy, and launching a professional campaign to know the real worth and status of anesthetists around us.

### Strengths & Limitations:

This study provides a comprehensive overview of both the statistical trends and personal reasons including powerful testimonials about stigma and dependency behind career choices in Baluchistan.

However, this study focused on a single institute i.e. PGMIQ. Different results may come up if it is carried out in multiple centers. Moreover, it is also expected that different results may be seen if the same study is conducted in other provinces. Aside from this another caveat is that this study focuses only on one group, i.e. those who have selected anesthesia as a career choice, however results can be different if studies are done on trainees in other specialties who did not opt for anesthesiology. These findings would give policymakers a clearer picture of why some medical fields are popular and others are not?

## CONCLUSION

This study revealed that the primary driving factor for selecting anesthesiology was the residents’ intrinsic fascination with the field, as well as their desire to pursue research and academic career.

### Author’s Contribution:

**MIK:** Literature search, concept of design, data acquisition, preparing draft of the manuscript.

**AK:** Data analysis and interpretation, Critical Review.

**AU:** Draft of paper, critical review,

All the authors have read and approved the final version. They are also accountable for the integrity of research.

## References

[1] Health Department Balochistan (2024). Govt of Balochistan [internet].

[2] Bhattacharya PK, Nair SG, Kumar N, Natarajan P, Chhanwal H (2021). Critical care as a career for anaesthesiologists. Indian J Anaesth..

[3] John M, Charles Z (2024). Anesthesiologist shortage in the United States:A call for action. J Med Surg Public Health..

[4] Al-Ansari SS, Khafagy MA (2006). Factors affecting the choice of health specialty by medical graduates. J Family Community Med..

[5] Kalimisetty S, Nelamangala K, Madhusudhana R (2023). Factors considered by Junior residents in selecting anesthesia as a career choice and stress level amongst the anesthesia postgraduates students:A questionnaire-based study. Indian J Clin Anesthesia.

[6] Nakayasu A, Kido M, Katoh K, Homma Y (2020). Survey on Specialty Preference and Work-Life Balance among Residents of Japanese Red Cross Hospitals. JMA J..

[7] Hohf-nova M, Hun-pacheco R, Muñoz-bustos D, Soto-carriel A, Pérez-villalobos C (2021). When it is time to decide:factors associated to the choice of a medical specialty. Rev Med Chil..

[8] Orbach-Zinger S, Rosenblum R, Svetzky S, Staiman A, Eidelman LA (2011). Attitudes to anesthesiology residency among medical students in the American and the Israel programs at Sackler Faculty of Medicine, Tel Aviv University. Isr Med Assoc J..

[9] Tyagi A, Kumar S, sethi AK, Dhaliwal U (2012). Factors influencing career choice in anesthesiology. Indian J Anesth..

[10] Khan J, Gilbert J, Sharma A, LeManch Y, Yee D (2015). Perspectives of anesthesia residents training in Canada on fellowship training, research and future practice location. Les perspectives des residents en anesthesia au Acnada pratique future. Can J Anesth..

[11] Bosco L, Gianni R, Lorello, Alana M, Flexman, Maya J (2020). Hastie. Women in anaesthesia:a scoping review. Br J Anaesth..

[12] Teddlie C, Tashakkori A Foundations of mixed method research:Integrating quantitative and qualitative approaches in the social and behavioral sciences. 2009. Los Angeles, CA:Sage.

[13] Izgi M, Basaran B, Ankay Yilbas A, Uzun S, Pamuk AG, Kanbak M (2019). Factors affecting the preference of anesthesia residents regarding subspecialty training. BMC Med Educ..

[14] Roberts LJ, Khursandi DC (2002). Career choice influences in Australian anaesthetists. Anaesth Intensive Care..

[15] Bissing MA, Lange EM, Davila WF, Wong CA, McCarthy RJ, Stock MC (2019). Status of women in academic anesthesiology:a 10-year update. Anesth Analgesia..

[16] Lorello GR, Cil T, Flexman AM (2020). Women anesthesiologists'journeys to academic leadership:a constructivist grounded theory-inspired study. Can J Anesth..

[17] Knoll MA, Griffith KA, Jones RD, Jagsi R (2019). Association of gender and parenthood with conference attendance among early career oncologists. J Am Med Assoc Oncol..

[18] Kandikar L, Vishwanath, Ghatapanadi S, E Khole SN, Balaraju TC (2019). Factors considered by final year MBBS students in selecting Anesthesia as a career Choice:A Questionnaire-Based Study. Indian J Anesth Analgesia..

[19] Chandran KV, Abraham SV (2020). Basic Life Support among Young Doctors in India. Indian J Crit Care Med..

